# Effects of work-interval duration and sport specificity on blood lactate concentration, heart rate and perceptual responses during high intensity interval training

**DOI:** 10.1371/journal.pone.0200690

**Published:** 2018-07-16

**Authors:** Diego Warr-di Piero, Teresa Valverde-Esteve, Juan Carlos Redondo-Castán, Carlos Pablos-Abella, José Vicente Sánchez-Alarcos Díaz-Pintado

**Affiliations:** 1 Research Institute, Faculty of Physical Activity and Sport Sciences, Catholic University of Valencia San Vicente Mártir, Valencia, Spain; 2 School of Doctorate, Catholic University of Valencia San Vicente Mártir, Valencia, Spain; 3 Department of Music, Plastic and Body Expression, Faculty of Education, University of Valencia, Valencia, Spain; 4 Faculty of Physical Activity and Sport Sciences, University of León, León, Spain; Norwegian University of Science and Technology, NORWAY

## Abstract

The aim of this study was to examine the impacts on blood lactate concentration, measured heart rate and assessment of perceived exertion during split sessions of equal relative load, as also their relationship to the specific sport practised. Nineteen regional-level athletes (nine middle and long-distance runners (cyclic group) and ten field-sport team players (acyclic group)) performed four high-intensity interval training (HIIT) sessions with work-interval durations of 10 s, 50 s, 90 s and 130 s. The sessions were carried out at their usual training sites with a separation of at least 48 hours. Blood lactate concentration was measured at rest and 3 min after the completion of each protocol. Heart rate was measured continuously during all sessions with a sampling rate of 1 s, and rating of perceived exertion (RPE) was requested at the end of the trial. The results showed an increase in blood lactate concentration, peak heart rate and rating of perceived exertion during long protocols as compared with short ones. No differences were observed in dependent variables between cyclic and acyclic groups. Significant but moderate correlations were observed between post-exercise blood lactate concentration, peak heart rate and RPE.

## Introduction

High -intensity interval training (HIIT) is defined as a method made up of repeated execution of bouts of exercise of an intensity equal to or above the lactate threshold, performed over shorter to longer periods intercalated with recovery phases that allow full or partial recovery [1–3]. The development of programmes that include HIIT, leads to significant increases in physiological variables when compared with continuous low-intensity activities [4, 5]. It has been reported that during intermittent runs a greater amount of time at VO_2max_ is spent with lower blood lactate responses (though not significantly so), indicating a more efficient work-cost rate in comparison with continuous running [6]. Further research [7] found that VO_2peak_ and %VO_2max_ were higher in the interval mode as compared with the continuous, but significantly higher post-exercise blood lactate was reported. Hence, an accurate determination of the impact of HIIT, as an external load, is a relevant factor in predicting different acute responses (internal load) and consequent functional adaptations in interval protocols [8–10].

In setting up a given intermittent exercise, the main components that can be manipulated are workload duration, workload intensity, recovery load, recovery duration and mean workload [1, 11]. Several studies have applied different configurations of these components in order to compare responses among HIIT protocols. Significant differences were found in physiological variables and total performance for intensities ranging from 110% to 140% of the maximum aerobic speed (MAS) during all-out intermittent runs [12]. Further evidence for this is supplied by Wakefield and Glaister [13], who reported a decrease in the time to exhaustion, with higher blood lactate concentrations, in a 115% MAS trial as against 105%. In addition, this study revealed that the time spent at or above 95% of VO_2max_ was longer for work intervals of 30 s than for 20 s and 25 s, with an interaction between exercise intensity and work interval duration. When protocols that are not all-out are applied (and consequently the total volume can be fixed in advance), the work interval duration and work-recovery ratio would influence the response to exercise. On these lines, it was found that when a total distance of 2400 metres was covered at 100% of maximal aerobic speed, longer work interval durations (30 s and 60 s) and work-recovery ratios (2:1 and 4:1) brought about a significantly greater percentage of VO_2max_ with higher values of rating of perceived exertion (RPE) and blood lactate concentration than with a shorter interval of 15 s and a ratio of 1:1 [14]. Another study [15] monitored both the volume (20 min) and the work-recovery ratio (1:1.5) to compare the effect of work duration on physiological and perceptual responses during HIIT at 120% of maximal aerobic speed. The results showed a greater blood-lactate concentration and RPE for a 24:36 s trial as against 6:9 s. Furthermore, the effect of work duration was also investigated for longer intervals extending from 1 min to 6 min under self-paced conditions and with a 1:1 work-recovery ratio [16]. The results showed that in sessions with a 1 min interval a significantly lower %VO_2_ value was elicited and a higher blood lactate was measured versus all the longer intervals of 2 min, 4 min and 6 min.

The prediction of physiological and perceptual responses related to intensity and work-recovery duration for precise combinations of HIIT has been investigated. However, the influence of the work-interval duration component in HIIT sessions remains unclear. In addition, the effects of work-interval duration and its relationship with specific sports, has not yet been investigated. Thus, the aim of this study was twofold: a) to examine the physiological and perceptual responses for different work-interval durations in HIIT sessions; and b) to determine differences between groups related to the sports discipline practised. It was hypothesized that maintaining constant all other HIIT load components, longer work-interval durations would elicit higher blood lactate concentration values and heart rate and perceived exertion responses, than shorter work-interval duration exercises. It also was hypothesized that, considering the type of exertion performed in competition, track and field athletes would tolerate long-interval duration formats better than team sport players, and team sport players would tolerate short-interval durations better than track and field athletes.

## Materials and methods

### Experimental overview

All the subjects completed a total of six sessions over a maximum period of four weeks. A separation of at least 48 hours from the last session or participation in any competition was implemented. The first and second sessions were used for Preliminary Testing in which maximal aerobic speed and time to exhaustion were determined. On the basis of these data, four experimental HIIT sessions with equal total volumes of effort, but different work-interval durations (S = 10 s, IS = 50 s, IL = 90 s, L = 130 s) were programmed for each participant. These training sessions were then executed in a random but counterbalanced order during Sessions 3 to 6. Comparisons of blood lactate concentration, heart-rate derived variables and perceptual responses were analysed in respect of the training sessions with different work-interval durations.

### Participants

Nineteen athletes (twelve male and seven females) volunteered to participate in the study. They all held individual sports licences as needed for participation in regional-level competitions regulated by Spanish sports federations. All subjects provided informed consent in writing before any participation. In addition, the participants completed a questionnaire, in which they recorded their experience of sports and current training programmes. Details of participants are given in Table 1. The research was approved by the Ethical Research Committee of the Catholic University of Valencia San Vicente Mártir. The inclusion criteria adopted were: (a) a minimum of five years in the specific sport practised, (b) attaining a total training frequency at least of three sessions per week, including any competitions, (c) having suffered no injuries during the three months before the investigation. In accordance with the characteristics of the sport practised, but only for the purpose of data analysis, subjects were classified into two groups: Cyclic Disciplines (n = 9), including middle- and long-endurance track and field sports (from 3000 m to Marathon), and Acyclic Disciplines (n = 10) comprising team field sports (football, n = 4; rugby n = 3; hockey, n = 3). This distinction was based on the hypothesis that different HIIT responses might be observed because of specific sports practised, in view of the type of exertion with which the subjects were familiar. Cyclic discipline athletes perform long-duration efforts in a continuous manner, whilst acyclic discipline athletes perform short efforts repeatedly throughout the duration of a competition. Hence, it might be expected that long HIIT activities would be better tolerated by cyclic track and field athletes. Conversely, short HIIT prescriptions with a larger number of accelerations might represent less stress for acyclic team sport players.

**Table 1:** Physical data and training experience of the participants.

	Cyclic (n = 9)	Acyclic (n = 10)
Age (y)	32.0 ± 12.0	23.2. ± 4.0
Height (cm)	170.7 ± 7.3	170.0 ± 10.0
Mass (kg)	63.9 ± 9.5	66.8 ± 12.0
Body fat (%)	11.2 ± 5.4	14.1 ± 4.9
HR_max_ (b.min^-1^)	187.7 ± 13.0	196.0 ± 8.5
VO_2 max_ (ml.kg^-1^.min^-1^)	59.2 ± 6.9	50.0 ± 6.6
Maximal Aerobic Speed (m.s^-1^)	4.6 ± 0.6	3.9 ± 0.5
Time to Exhaustion (s)	454.3 ± 103.3	300.3 ± 70.4
Distance to Exhaustion (m)	2118.7 ± 621.3	1174.6 ± 374.1
Length of training (y)	10.6 ± 8.0	7.8 ± 4.1
Frequency (sessions.week^-1^)	5.9 ± 1.3	4.2 ± 0.6

Values are expressed as mean ± SD.

HR_max_ = maximal heart rate recorded in preliminary testing.

### Procedures

All testing was carried out at the athletes´ usual training sites. The sessions started with a standardized warm-up consisting of 5 min of continuous running at 60% of MAS, followed by 3 min of free movements (hopping, skipping, or running backwards). Before tests started in Sessions 2 to 6, a preparatory bout of 30 s at MAS was performed by the subjects to adjust their pace, followed by a 3 min active recovery period. Heart rate (HR) was recorded continuously throughout every session using a heart rate monitor (RS800CX, Polar Electro Oy, Kempele, Finland) with a sampling frequency of 1 s. The data were then transferred to PolarProtrainer 5 software (Polar Electro, Kempele, Finland). At rest, and 3 min after the cessation of exercise, capillary blood samples were obtained from the subjects´ earlobe to determine lactate concentration using a portable lactate analyser (Lactate Pro 2, Arkray, Japan). RPE was monitored in the four experimental trials using the CR-10 scale [17]. Immediately after the end of the final bout, subjects were asked to indicate their perceived exertion for the session completed. The answer could be given either as a number or a category, but these data were recorded numerically in all cases, numbers being assigned to categories.

#### Preliminary testing

In the first session, before the standardized warm-up protocol was begun, all subjects were weighed and their heights were measured (Seca 704 s, Hamburg, Germany). Skinfold thickness values were obtained (Holtain caliper, Holtain Ltd.) [18, 19]. Thereafter, Multi-stage Shuttle Run Tests [20] were performed in pairs or individually. These tests have been shown to be valid for an indirect assessment of maximum oxygen uptake. The tests are described as continuous, incremental and maximum and involved running between two parallel lines 20 m apart, in response to a sound signal that indicated the pace. The starting speed was set at 8.5 km/h, and increased by 0.5 km/h each 1 min stage. The test ended when subjects stated they were exhausted or continued running but failed in reach the line three times in one stage. The final stage when fully completed was recorded as the maximum. The speed achieved in this stage was then adjusted using Eq 1 and the maximal aerobic speed for field activities was calculated [21].

$$MAS_{track}(km/h)=1.81*MAS_{shuttle}(km/h)-7.86$$ (1)

Where *MAS*_*track*_ is the maximal aerobic speed estimated for track use, and *MAS*_*shuttle*_ is the speed of the last stage completed.

In the second session, a Time to Exhaustion Test [12, 22] was executed individually on a track designed specifically for the purpose. The test required athletes to carry on running at their maximal aerobic speed for most of the time. To assist athletes in maintaining a constant speed, marks at equal separations were set along the track to indicate fractions of laps. Further secondary marks were placed ± 1 m from these partial marks, delimiting the zones considered valid for one lap. A digital watch (Geonaute W200M, Oxylane, Villeneuve d´Ascq cedex, France) was placed round the wrist of each subject and programed with a loop countdown that emitted a sound every 10 s. The subjects were instructed to be passing alongside marks (or at least to be within the zones) when a beep was heard. The distance between marks was set according to each subject´s MAS (e.g. for a maximal aerobic speed of 4.1 m/s, the corresponding distance between marks was 41 m). The total time in seconds was monitored by the researchers using a chronometer (HS-5, Casio Computer Co., Tokyo, Japan) and by recording the partial distances completed. The test was deemed to be over when a subject could no longer continue running or when two consecutive partials were not reached in time. The Total Time to Exhaustion and Total Distance to Exhaustion where then determined, relative to the last partial completed.

#### Experimental trials

Four HIIT sessions of equal total volume and intensity were programmed for each participant. In order to ensure equal loading for participants, the intensity was fixed at 100% of MAS and the total volume was equivalent to Distance to Exhaustion. A work-recovery ratio of 1:1 was applied to all the sessions. Thus, the total session duration for each experimental trial was double the Time to Exhaustion. Work-interval duration varied among protocols, ranging from 10 s to 130 s with intervals of 40 s. The different protocols based on the length of bouts of exercise are shown in Table 2. If any given trial did not match the mean assigned for the protocol, it was adjusted using Eq 2:

$$t_{rep}(s)=T_{\text{lim}}/a$$ (2)

Where *t*_*rep*_ is duration in seconds of each repetition, *T*_lim_ is the total Time to exhaustion and (*a*) is the whole number that yields the value closest to the mean for the trial.

**Table 2:** Split trials based on work-interval duration.

TRIAL	Type of Intervals	Work Duration (s)	Recovery Duration (s)	Number of Repetitions	Repetition Distances (m)	Total Volume (m)
**S**	Short	10	10	37.0 ± 11.6	42.0 ± 6.4	1619 ± 688
**IS**	Intermediate short	50	50	7.0 ± 2.2	212.0 ± 34.7	1619 ± 683
**IL**	Intermediate long	90	90	4.0 ± 1.3	371.0 ± 1.3	1622 ± 693
**L**	Long	130	130	3.0 ± 1.0	545.0 ± 92.5	1614 ± 701

Values are expressed as mean ± SD.

During the execution of IS, IL and L trials, the same procedure used in the Time to Exhaustion test for marking the track and ensuring the pace was maintained was repeated. In the S trial the length of the track was the distance that subjects were required to cover in 10 s. Subjects ran from the start mark to the finish mark, remaining there for the rest period. Thereafter, they returned, running in the opposite direction. Subjects started every bout at a verbal order given after a 5 s countdown. After each bout ended the recovery period prior to the next bout was monitored by the researchers and indicated automatically by the watch countdown signal.

### Statistical analyses

The normality and homogeneity of variances were verified using the Shapiro-Wilk and Levene’s tests, respectively. Blood-lactate concentration values showed deviations from normality, so the data were transformed into natural logarithm format in order for parametric procedures to be used [23, 24]. All values are shown as mean and *SD*. The one-second heart-rate sampling details were converted into text files so as to calculate the heart-rate-derived variables for each trial, Peak HR being defined as the highest 10 s average recorded in one single trial, mean HR the mean of all of one trial (from the first second of the first repetition until the last second of the last recovery period) and recovery HR the difference between peak HR from the trial and the mean HR over a 5 s period that occurred 60 s after the test [25]. A two-way (trial x specific sport) repeated measures analysis of variance (ANOVA) was used to analyse the effects of trial length and specific sport on pre- and post-exercise blood-lactate concentrations, heart-rate-derived variables and assessments of perceived exertion. The power (1-β) and the effect size (η*p*^2^) were estimated for all of the ANOVAs. A post-hoc Bonferroni test was used to determine the differences reported in the ANOVAs. Correlations between peak heart rates, rating of perceived exertion and post-exercise blood-lactate concentrations were calculated using the Pearson correlation coefficient (*r*). For results to be considered significant, bilateral α level was set at p < 0.05 for all tests. All data analysis was conducted using the Statistical Package for Social Sciences (version 20.0, SPSS, Inc., Chicago, IL, USA).

## Results

### Blood lactate

**Fig 1:**
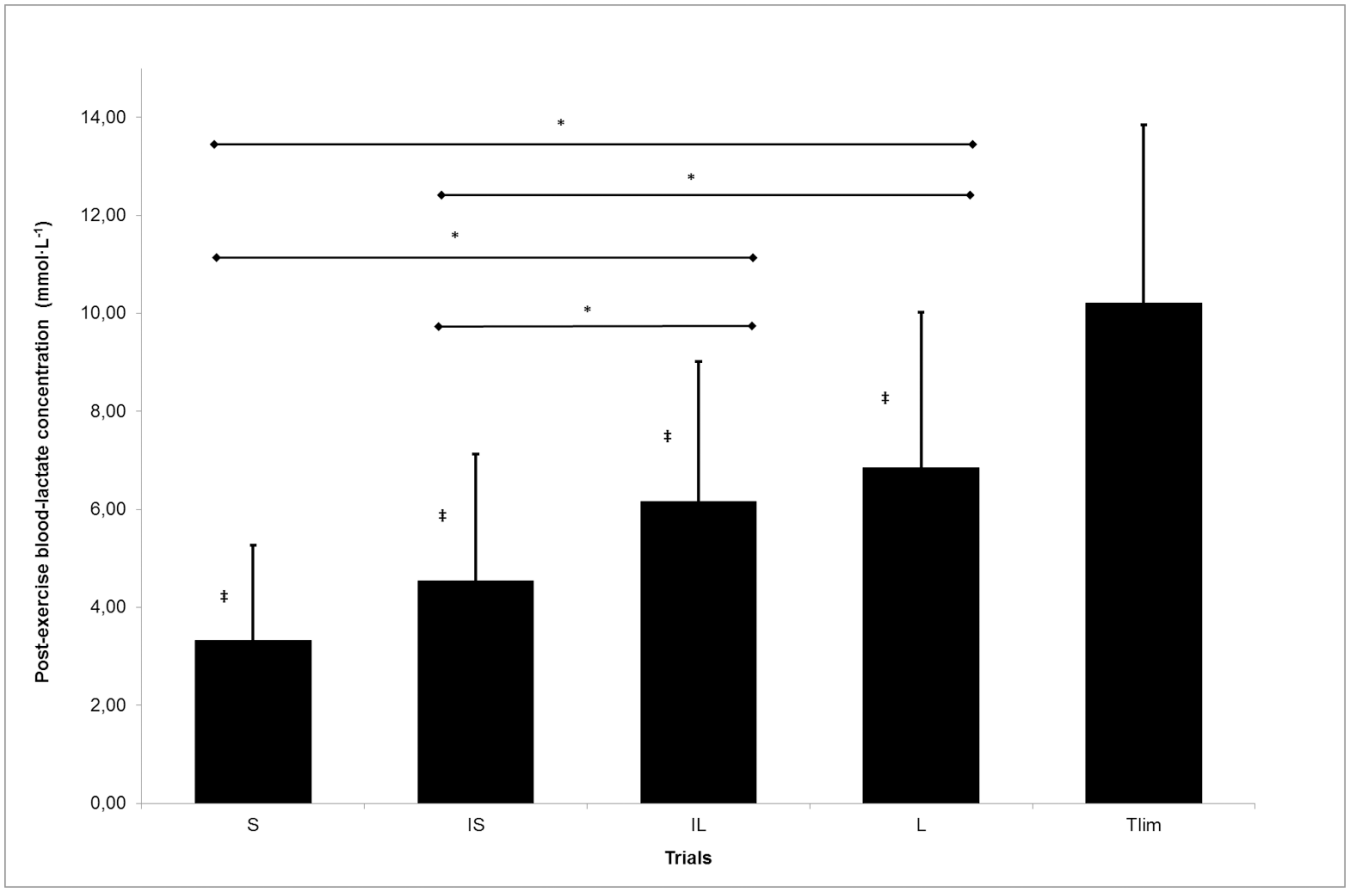
Mean pre- and post-exercise blood-lactate concentrations for the time-to-exhaustion test and the split trials. ⱡ indicates significant differences from Tlim (p < 0.01), * indicates significant differences (*p* < 0.01) among trials. Tlim = Time to Exhaustion.

A summary of the responses in blood-lactate concentrations for the Time-to-Exhaustion Test and the split trials based on work-interval duration is provided in Fig 1. No significant differences were found in pre-exercise blood-lactate concentration, irrespective of the protocol (*F*_(4,14)_ = 0.57; *p* = 0.69; power = 0.15; η*p*^2^ = 0.14). The repeated–measures ANOVA showed significant differences in post-exercise blood-lactate concentration between protocols (*F*_(4,14)_ = 33.73; *p* = 0.000; power = 1.00; η*p*^2^ = 0.91). The post-hoc analysis revealed that the Time-to-Exhaustion Test gave significantly higher values than all the split trials (*p* = 0.000). The Bonferroni test also indicated differences among trials, with the S trial having significantly lower figures than the IL (*p* = 0.000) and L (*p* = 0.000), and the IS trial significantly lower than the IL (*p* = 0.001) and L (*p* = 0.000). However, no significant interaction between protocol and specific sport was observed for post-exercise blood-lactate concentrations (*F*_(4,14)_ = 0.19; *p* = 0.941 power = 0.08; η*p*^2^ = 0.05).

### Heart-rate-derived variables

One major effect of trial duration was observed in peak HR (*F*_(3,15)_ = 31.59; *p* = 0.000; power = 1.00; η*p*^2^ = 0.86) with post-hoc analysis revealing a lower peak HR value in S than in IL (*p* = 0.004) and in L (*p* = 0.000), together with a lower value in IS than in IL and L (*p* = 0.000), as shown in Table 3. No significant interaction (protocol x specific sport) was observed for peak HR (*F*_(3,15)_ = 0.08; *p* = 0.96; power = 0.06; η*p*^2^ = 0.02). An effect of the trials was also observed in mean HR (*F*_(3,15)_ = 21.49; *p* = 0.000; power = 1.00; η*p*^2^ = 0.81), where the S trial had a significantly higher value than all the other trials (*p* = 0.001). No significant interaction (protocol x specific sport) was observed for mean HR (*F*_(3,15)_ = 3.22; *p* = 0.53; power = 0.62; η*p*^2^ = 0.39). There was no significant effect of trial duration (*F*_(3,15)_ = 2.88; *p* = 0.07; power = 0.56; η*p*^2^ = 0.36) or specific sport (*F*_(3,15)_ = 0.76; *p* = 0.53; power = 0.17; η*p*^2^ = 0.13) on Recovery HR.

**Table 3:** Mean data for heart-rate derived variables and rating of perceived exertion in the four split trials. Values are expressed as mean ± SD.

Dependent variables	S	IS	IL	L
HR_mean_ (% HR_max_)				
Cyclic	85.6 ± 3.3	80.4 ± 3.6 ^†^	78.2 ± 4.6 ^†^	78.4 ± 4.1 ^†^
Acyclic	84.5 ± 5.3	80.7 ± 4.6 ^†^	81.4 ± 5.2 ^†^	79.7 ± 5.0 ^†^
Overall	85.1 ± 4.4	80.5 ± 4.0 ^†^	79.9 ± 5.0 ^†^	79.1 ± 4.5 ^†^
HR_peak_ (% HR_max_)				
Cyclic	91.0 ± 3.6	91.2 ± 2.6	94.4 ± 3.2 ^†^ ^Ŧ^	95.7 ± 3.1 ^†^ ^Ŧ^
Acyclic	89.9 ± 4.1	90.5 ± 2.9	94.0 ± 2.7 ^†^ ^Ŧ^	95.4 ± 2.1 ^†^ ^Ŧ^
Overall	90.5 ± 3.8	90.9 ± 2.7	92.2 ± 2.9 ^†^ ^Ŧ^	95.5 ± 2.6 ^†^ ^Ŧ^
HR_rec_ (% decrease)				
Cyclic	21.4 ± 4.5	27.4 ± 6.7	29,8 ± 12.7	25.5 ± 10.9
Acyclic	20.9 ± 5.3	23.7 ± 8.2	22.4 ± 10.0	21.1 ± 6.7
Overall	21.5 ± 4.8	25.8 ± 7.6	26.7 ± 11.7	23.1 ± 9.0
RPE				
Cyclic	2.5 ± 0.9	2.7 ± 0.5	3.9 ± 1.1 ^†^ ^Ŧ^	4.0 ± 1.6 ^†^ ^Ŧ^
Acyclic	2.9 ± 1.1	3.2 ± 1.4	4.5 ± 1.4 ^†^ ^Ŧ^	5.5 ± 2.1 ^†^ ^Ŧ^
Overall	2.7 ± 1.0	2.9 ± 1.1	4.2 ± 1.3 ^†^ ^Ŧ^	4.8 ± 2.0 ^†^ ^Ŧ^

Note: HR_mean_ = Mean heart rate for the session; HR_peak_ = Peak heart rate; HR_rec_ = Recovery heart rate; % HR_max_ = Percentage of maximal heart rate; RPE = rating of perceived exertion.

^†^ Significantly different (p < 0.05) from S.

^Ŧ^ Significantly different (p < 0.05) from IS.

### Rating of perceived exertion

There were significant differences in RPE from trial to trial (*F*_(3,15)_ = 25.64; *p* = 0.000; power = 1.00; η*p*^2^ = 0.60). Post hoc analysis showed a significantly greater value for L than for S (*p* = 0.000) and IS (p = 0.000). Likewise, there was a significantly greater value for IL than for S (*p* = 0.000) and IS (*p* = 0.000), as shown in Table 3. No interactive effect (protocol x specific sport) was observed on mean RPE (*F*_(3,15)_ = 1.97; *p* = 0.16; power = 0.35; η*p*^2^ = 0.10).

A significant correlation was observed between post-exercise blood lactate concentration and RPE (*r* = 0.60; *p* = 0.000). A moderate correlation was also observed between blood lactate concentration and peak HR (*r* = 0.62; *p* = 0.000).

## Discussion

The first aim of this study was to examine the influence of work-interval duration on blood lactate concentration, heart rate measured and rating of perceived exertion, during HIIT sessions of equal total volume and intensity. Differences in blood lactate concentration were observed between short and long exercises. However, no differences were observed when comparing short intermittent protocols (S versus IS) and long interval protocols (IL versus L). A similar response was observed for rating of perceived exertion. Furthermore, heart rate measurements seemed to be sensitive to 40 s interval variations, showing significant differences between trials. The second aim of the study was to determine whether the specific sport practised might elicit different responses, during HIIT with differing work-interval durations. No differences were observed between cyclic and acyclic groups in blood lactate concentration, heart rate and rating of perceived exertion.

It would appear that no studies have analysed the responses occurring in HIIT sessions of equal relative load, over a wide range of work-interval durations running from very short (10 s) to long (above 2 min) exercises. The volume stablished for each subject was the total distance covered in the Time to Exhaustion Test. In the present study, the mean total time performed in this test was 373 ± 116 s with a mean post-exercise blood lactate concentration of 10.2 ± 3.6 mmol/L, these values being very similar to those reported by Dupont, Blondel, Lensel and Berthoin, which were 362 ± 109 s and 10.3 ± 3.5 mmol/L, respectively [12]. As expected, blood-lactate concentrations in the continuous trial were significantly higher than in all the split protocols. Previous studies [6, 7] have compared the responses in blood-lactate concentration between continuous and interval protocols. In one study [7] a significantly higher blood-lactate value was noted during the interval exercises. A possible explanation for this difference might be variation in the intensity applied to the different protocols, as result of an equal-effort criterion based on RPE, leading to lesser intensity in the continuous bout. Other authors [6] reported no differences in blood-lactate concentrations between continuous and intermittent formats. This might be explained both by differing intensities applied to the various protocols and by the all-out format undertaken in sessions. This involved differences in the total distances covered by a given subject during the various trials.

The findings being reported here, confirming our first hypothesis, suggest that if all the other training-load components are kept constant during split sessions, longer trials elicit a greater stimulation of the glycolytic pathways for energy production, reflected in higher values for blood-lactate concentrations. This is in agreement with previous studies [10, 13–15] which gave support for the notion that longer work duration substantially increases anaerobic metabolism in HIIT. On the other hand, the low values observed for the S and IS trials may indicate a predominantly utilization of oxygen sources bound to myoglobin supplies [26]. However, when work-interval variation is accompanied by manipulation of other training components such as work-to-rest ratio, intensity, or total volume, different lactate responses can be observed. For example, Wakefield and Glaister [13] reported significant differences in lactate values in all-out trials with work durations that ranged from 20 s to 30 s. In the current study, the extension of work- interval duration from 10 s to 50 s has not been sufficient to increase significantly blood lactate values, however, blood lactate concentration values increased significantly since work-interval duration of 90 s. In a study conducted by Rozenek, Funato, Kubo, Hoshikawa and Matsuo work intervals of 30 s and 60 s showed a significant increase in blood-lactate concentrations as compared with a 15 s interval, in short intermittent runs at 100% vVO_2 max_. This is in partial contrast with the present results which showed no differences in lactate responses during short intermittent trials. A possible explanation might be variations in the work-to-rest ratio. Recovery periods were fixed at 15 s and consequently the work-to-rest ratio increased over the course of trials. In addition, the values reported in the study quoted were higher than in the current work, even for the 15:15 trial, possibly as result of the total volume of 2400 m completed, representing 1.5 times the volume achieved in the time-to-exhaustion test.

Using a fixed total volume and work-to-rest ratio, Price and Moss [15] observed that lactate values in a longer protocol (24 s of work to 36 s of recovery) were significant greater than in a shorter protocol (6 s of work to 9 s of recovery), during supramaximal runs at 120% vVO_2max_. Despite the higher intensity performed in that study, blood-lactate values were similar to those observed in the present work for the S and IS intervals, possibly as result of the longer work-to-rest ratio of 1:1.5 applied. Other authors [7] have investigated the variations in responses between 30:30 s and 120:120 s protocols during high-intensity cycling, reporting no significant differences in post-exercise lactate concentrations. However, exercise intensity varied from 110% power output for the 30 s trial to 95% power output in the 120 s trial. In their investigation the lactate value reported for the 120 s protocol was 10.7 ± 2.8 mmol/L, this being higher than the response observed for the L trial in the current study (≈ 6.9 mmol/L). Light might be shed on the difference by the all-out format implemented in the study, requiring near maximal glycolytic stimulation. Seiler and Sjursen [14] also analysed lactate responses in long interval protocols from 1 min to 6 min of work duration at a self-paced intensity, reporting similar blood lactate concentrations of ≈ 4.5 mmol/L across their protocols. Nevertheless, a decrease in running speed was observed as interval duration grew, with lactate values remaining constant over the four experimental conditions.

Work Interval duration also affected the responses in HR derived variables. Peak HR reached a minimum of 90% of HR_max_ during all trials, which may be considered sufficient to stimulate cardiovascular adaptations [5, 25]. However, a higher HR peak was observed in the long exercises as compared with the short ones. Mean HR in the S trial was significantly higher than in all other trials, as consequence of the reduced time available for achieving any significant decrease in HR during the recovery period. In fact, an increase in HR was observed in recovery phases in this trial. The value of 85% HR_max_ registered in the S trial was similar to those noted in previous studies for comparable durations [7, 10, 14]. The results being reported here are consistent with fact that during HIIT a smaller oscillation around mean HR is expected [11] and that a higher mean HR need not necessarily be accompanied by high lactate values [1, 11].

RPE responses were similar to blood-lactate concentrations and HR peaks in relating to the length of the work interval. In other words, values in short intermittent trials were significantly lower than in long interval trials, with no significant differences between S and IS or between IL and L. Participants rated short and long trials as moderate and strong, respectively. This perception is in agreement with those noted in previous studies with similar intervals set [14, 15]. It has been stated that RPE methods may provide a simple tool for self-regulating exercise intensity during steady-state and interval formats [8, 27]. However, they have some limitations in determining precise manipulations of physiological responses in HIIT [1]. In the current study, training intensity was established on the basis of performance measurements. Hence, RPE was used as a monitoring variable for a given session format and not to adjust exercise intensity.

The distinction of groups was based on the hypothesis that different HIIT responses would be observed due to sport specificity, considering the type of exertion that the subjects were familiarized with. Cyclic discipline athletes perform long duration efforts in a continuous manner while acyclic discipline athletes perform short efforts repeatedly along competition. Therefore, we had expected that long HIIT prescriptions would be better tolerated by cyclic track and field athletes. Conversely, short HIIT prescriptions with a higher number of accelerations would represent a lesser stress to acyclic team sport players. Our results showed that there was no effect of sport specificity on exercise responses, thus we rejected the second hypothesis. A possible explanation for this fact may be the low total volume performed by the participants, thus, further research is needed to examine whether a higher total volume may elicit different responses related to sport specificity. In addition, team sport players are habituated to execute short bouts of exercise during competitions that also include other mechanical components such as changes of direction and decelerations. Further studies should contemplate this factor in order to determine the optimal HIIT load appropriated to a specific discipline.

## Conclusions

The data obtained in this study suggest that if all other training load components are kept constant, the duration of the work interval may produce different stresses even if individualized protocols are applied. Short intermittent formats (below 60 s) allow a given session to be completed with lower values for blood-lactate concentrations and a lesser perception of the effort, whereas long interval formats elicit greater participation by the glycolytic pathway. No different responses associated with specific sports practised were observed in the split trials. Thus, in view of the importance of targeting specific adaptations, coaches could use the duration of the work interval as a tool to enable the orientation of HIIT sessions towards the objectives set, whether for track and field athletes or for team game players.

## Practical applications

Our design was orientated to a practical approach in HIIT prescription. For this reason, all tests were conducted in the athletes’ usual training sites and data were collected using minimal but reliable devices that were well kwon by trainers and participants. These devices did not interfere with exercise performance due to their simple application and to the fact that all participants had used them several times along their training programmes. Their use allowed to obtain sensible data avoiding any distortion of their normal running technique or training session development that could be caused by the use of more complex devices (e.g. gas analyzer). The participants were experienced athletes of regional-level, who undertook a minimum of three sessions per week. Thus, the results and interpretation of the data should be related to this population if it would be used as a specific monitoring tool to analyze the responses expected to similar HIIT prescriptions in future training sessions. Laboratory assessment’s devices, such as near infrared spectrometry or gas analyzers, would be necessary for a deeper interpretation of the metabolic sources related to the different interval durations.

## Supporting information

S1 Fig Heart rate data of one of the participants during the four fractionated trials. (TIF)

S1 Appendix Sport experience questionnaire Spanish version. (PDF)

S2 Appendix Sport experience questionnaire English version. (PDF)

S3 Appendix Minimal anonymized dataset. (XLSX)
