# Supplementary figures and images for: Effects of work-interval duration and sport specificity on blood lactate concentration, heart rate and perceptual responses during high intensity interval training
Source: PLoS One. 2018 Jul 16;13(7):e0200690. doi: 10.1371/journal.pone.0200690 (PMC6047801; doi:10.1371/journal.pone.0200690)

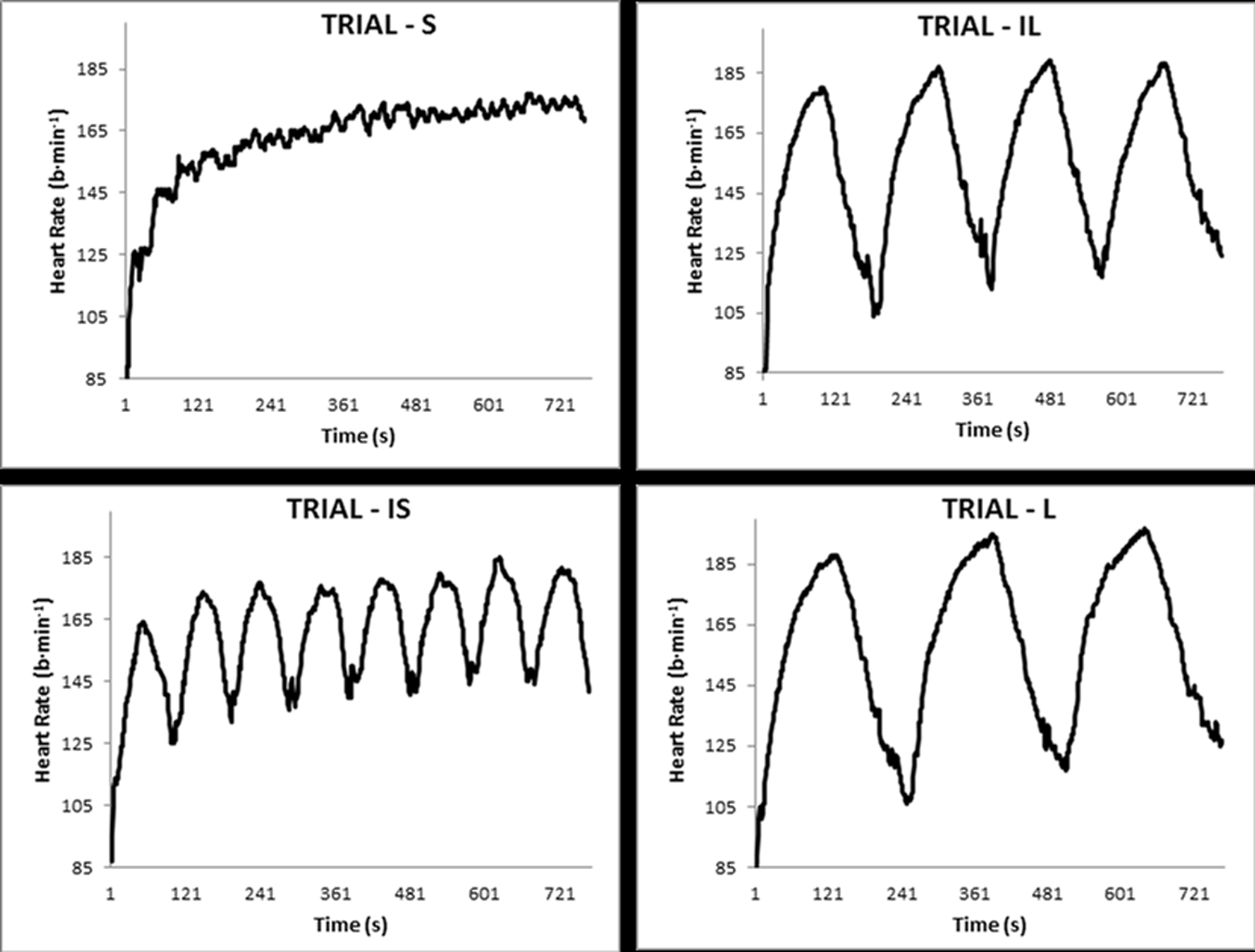

Supplement: S1 Fig — (TIF) [file pone.0200690.s001.tif]
