# Supplementary material for: Effects of work-interval duration and sport specificity on blood lactate concentration, heart rate and perceptual responses during high intensity interval training
Source: PLoS One. 2018 Jul 16;13(7):e0200690. doi: 10.1371/journal.pone.0200690 (PMC6047801; doi:10.1371/journal.pone.0200690)
Supplement: S1 Appendix — (PDF) [file pone.0200690.s002.pdf]

Universidad Católica de Valencia San Vicente Mártir

Título del Estudio: "Efectos de la duración del intervalo de trabajo y la especificidad deportiva sobre el lactato sanguíneo, la frecuencia cardíaca y la percepción del esfuerzo durante entrenamientos intervalados de alta intensidad".

Investigadores: Warr di Piero, D., Valverde Esteve, T.; Redondo-Castán, JC.; Pablos Abella, C.; Sánchez-Alarcos Díaz-Pintado, J.V.

CUESTIONARIO DE ANTECEDENTES DEPORTIVOS Y RÉGIMEN DE ENTRENAMIENTO

APELLIDOS Y NOMBRE: ____

DNI: ________________ Teléfono de contacto: ________________

Fecha Nacimiento: ___ /___ /___ SEXO: H – M

Disciplina Deportiva: ______________ Especialidad: ____________ División/Categoría: ______________

Período actual de entrenamiento: pre-competitivo / competitivo / transición

Frecuencia de entrenamiento (sesiones / semana): __________

1. ¿Cuántos años hace que practica su disciplina de manera federada? __________________

2. ¿Ha tenido algún tipo de lesión en los últimos seis meses, que lo haya obligado a suspender la práctica deportiva durante un período prolongado (más de 10 días)? En caso afirmativo, especificar.

   SI ☐ NO☐ ____

3. ¿Lleva una dieta adecuada a la práctica deportiva y/o un control nutricional? Especificar.

   SI ☐ NO☐ ____

4. ¿Utiliza sistemas de facilitación de la recuperación entre entrenamientos? Especificar.

   SI ☐ NO☐ ____

5. ¿Ingiere algún tipo de suplemento nutricional o complemento vitamínico? Especificar.

   SI ☐ NO☐ ____

6. Ingiere algún medicamento de manera habitual? Especificar.

   SI ☐ NO☐ ____
