# Supplementary material for: Effects of work-interval duration and sport specificity on blood lactate concentration, heart rate and perceptual responses during high intensity interval training
Source: PLoS One. 2018 Jul 16;13(7):e0200690. doi: 10.1371/journal.pone.0200690 (PMC6047801; doi:10.1371/journal.pone.0200690)
Supplement: S2 Appendix — (PDF) [file pone.0200690.s003.pdf]

Title: "Effects of work-interval duration and sport specificity on blood lactate concentration, heart rate and perceptual responses during high intensity interval training".
Researchers: Warr di Piero, D., Valverde Esteve, T.; Redondo-Castán, JC.; Pablos Abella, C.; Sánchez-Alarcos Díaz-Pintado, J.V.

QUESTIONNAIRE OF SPORT EXPERIENCE AND CURRENT TRAINING

SURNAMES AND NAME: ____

Id NUMBER: ______________ Contact phone: ______________

Birth Date: ___ /___ /___ SEX: F – M

Sport Discipline: ____________ Speciality: _____________ Division/Category: _______________

Current training period: pre-season / competitive / inter-season transition

Actual training frequency (sessions / week): _________

1. How long have you been practising your disicipline in a competitivelly? ________________

2. During the last three months, have you suffered any injured that force you stop your normal training for more than 10 days? If affirmative, please comment.

   YES ☐ ☐ NO ____

3. Do you maintain a diet accord to sport practice and/or nutritional supervision? Comment.

   YES ☐ ☐ NO ____

4. Do you use any recovery facilitation system between training sessions? Comment

   YES ☐ ☐ NO ____

5. Do you take any dietary supplementation? Comment.

   YES ☐ ☐ NO ____

6. Do you take any medicine regularly? Comment.

   YES ☐ ☐ NO ____
